# Tunneling, cognitive load and time orientation and their relations with dietary behavior of people experiencing financial scarcity – an AI-assisted scoping review elaborating on scarcity theory

**DOI:** 10.1186/s12966-024-01576-9

**Published:** 2024-03-04

**Authors:** Annemarieke van der Veer, Tamara Madern, Frank J. van Lenthe

**Affiliations:** 1 Research Group of Debt and Debt Collection, University of Applied Sciences Utrecht, Utrecht, PO Box 85397, 3508 AJ The Netherlands; 2 Department of Public Health, Erasmus MC University Medical Center Rotterdam, PO Box 2040, Rotterdam, 3000 CA The Netherlands

**Keywords:** Dietary behavior, Financial Scarcity, Tunneling, Time Orientation, Cognitive Load, Self-Control

## Abstract

**Background:**

The concept of a financial scarcity mindset has raised much attention as an explanation for poor decision-making and dysfunctional behavior. It has been suggested that financial scarcity could also impair dietary behavior, through a decline in self-control. Underlying cognitive mechanisms of tunneling (directing attention to financial issues and neglecting other demands), cognitive load (a tax on mental bandwidth interfering with executive functioning) and time orientation (a shift towards a present time horizon, versus a future time horizon) may explain the association between financial scarcity and self-control related dietary behavior. The current scoping review gathers recent evidence on how these mechanisms affect dietary behavior of people experiencing financial scarcity. It builds on a theoretical framework based on insights from behavioral economics and health psychology.

**Methods:**

A literature search was executed in six online databases, which resulted in 9.975 papers. Search terms were tunneling, cognitive load and time orientation, financial scarcity, and dietary behavior. Screening was performed with ASReview, an AI-ranking tool. In total, 14 papers were included in the scoping review. We used PRISMA-ScR guidelines for reporting.

**Results:**

Limited evidence indicates that a scarcity mindset could increase tunneling, through attentional narrowing on costs of food, which then directly impacts dietary behavior. A scarcity mindset involves experiencing financial stress, which can be understood as cognitive load. Cognitive load decreases attentional capacity, which could impair self-control in dietary choices. Financial scarcity is related to a present time orientation, which affects dietary choices by shifting priorities and decreasing motivation for healthy dietary behavior.

**Conclusions:**

A scarcity mindset affects dietary behavior in different ways. Tunneling and a shift in time orientation are indicative of an attentional redirection, which can be seen as more adaptive to the situation. These may be processes indirectly affecting self-control capacity. Cognitive load could decrease self-control capacity needed for healthy dietary behavior because it consumes mental bandwidth. How a changing time orientation when experiencing financial scarcity relates to motivation for self-control in dietary behavior is a promising theme for further inquiry.

## Background

### Introduction

Dietary behavior inconsistent with recommendations for healthy diets (further referred to as ‘healthy dietary behavior’), such as eating too much high caloric foods, and too little fruits and vegetables, is more prevalent among people with low socioeconomic positions [1, 2] and is widespread in high income countries in Europe and the US [3, 4]. Several factors have been appointed as drivers for these inequalities in dietary behavior, such as (perceived) higher costs of healthy foods [5–9], stress and poor sleep due to economic constraints [10–12], or living in a socio-economically deprived area [13].

### The scarcity theory

In 2013 Mullainathan and Shafir introduced the concept of financial scarcity mindset, which can be defined as an alteration of the way people think and act as a result of the feeling of having too little financial means to make ends meet. It may lead to counterproductive decision making through tunneling (directing attention towards what is scarce and neglecting other information), cognitive load (which taxes available cognitive bandwidth, impairing executive functions) and a shift in time orientation towards being more present oriented versus more future oriented [14]. A scarcity mindset could disrupt self-control exertion because it consumes limited cognitive bandwidth that is also necessary to abstain from temptations and to focus on long-term goals [15–20]. Empirical evidence that financial scarcity is negatively related to self-control is building [21, 22], although self-control was not always reduced in economic scarce conditions [23, 24].

Self-control capacity contributes to diet quality, because exerting self-control helps people to abstain from eating palatable foods [25–38]. Self-control is defined as ‘the capacity to handle dilemmas, between pursuing longer-term goals over instant gratification, by choosing and acting upon a larger but delayed reward over a smaller but sooner one, therefore delaying gratification’ [39, 40]. Although the heritability of self-control is as much as 60% [41] self-control is not entirely static. Exerting self-control can be more difficult due to e.g., lack of sleep [42] a high cognitive load [43, 44] or processes that interfere with motivation or attention [31]. Self-control involves several processes, such as initiating plans and actions to pursue desired goals and ignoring or restraining distracting impulses [45, 46].

It has been suggested that financial scarcity is also related to dietary choices that are inconsistent with recommendations for health [4, 24, 47–49]. A recent study among Dutch adults reported a negative association between financial scarcity and dietary quality. Further, the variance in dietary quality was better explained by a model of the Theory of Planned Behavior including food insecurity and/or financial scarcity [50]. Self-control may affect this association [10, 17, 18, 51–55]. However, empirical evidence is scarce and the limited studies that have tested the hypothesis that financial scarcity impairs dietary behavior through a declined self-control capacity found that the mediating role of self-control is limited in size [22] and different for women and men [56].

The scarcity theory states that the cognitive mechanisms of tunneling, cognitive load and time orientation explain when, how and why self-control failures occur [14] and it is widely accepted that self-control capacity is needed for healthy dietary behavior [31]. However, to what extent and how these mechanisms explicitly or differentially influence the dietary choices of people experiencing financial scarcity has hardly been studied. A few recent reviews touch upon the scarcity mindset and offer a first glimpse of how these processes may operate. Laraia, Leak, Tester & Leung [11] and Kraft & Kraft [55] explored the role of biobehavioral (e.g. stress and lack of sleep, which affect hormonal and immune responses), and psychological factors (e.g. cognitive load and time orientation eliciting self-control failures) affecting diet quality of people with low incomes [11, 55]. Others demonstrated that financial scarcity affects neural processes involved in goal-directed decisions concerning the willingness to pay for familiar food items [57].

To further advance the understanding how a scarcity mindset may impair dietary behavior, a scoping review was conducted to systematically explore the available evidence on the associations between tunneling, cognitive load and time orientation respectively, and dietary choices of people in a scarcity mindset. By focusing on a financial scarcity mindset, we go beyond socioeconomic positions in society. We take ‘the subjective experience of having too little financial resources’ [14] as a central starting point and synthesize current evidence of the role of tunneling, cognitive load and time orientation in dietary behavior of people experiencing financial scarcity.

### Theoretical framework

**Fig. 1:**
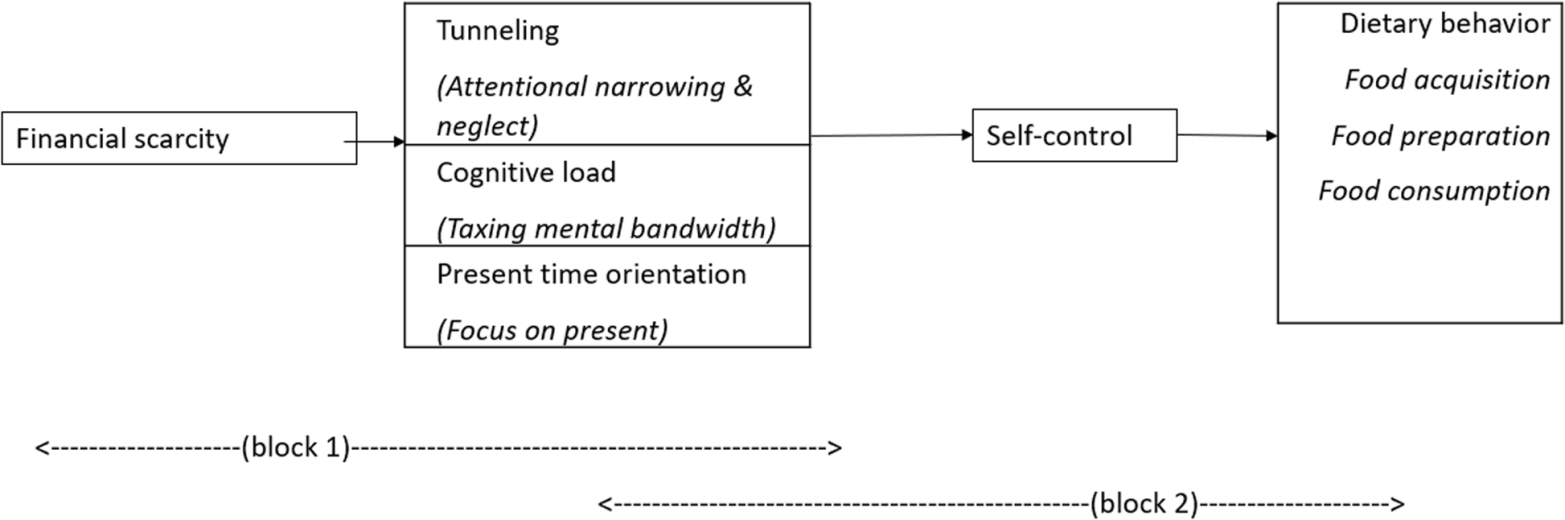
Theoretical framework applying scarcity theory to self-control related dietary behavior

The scoping review builds on a theoretical framework which integrates what is already known about how, among adults, financial scarcity affects self-control through tunneling, cognitive load and time orientation (visualized in Fig. 1 as block 1), and how these processes affect self-control in the context of dietary behavior (block 2). We connect these bases of evidence which are grounded in behavioral economics and health psychology and conclude our framework with premises about how dietary behavior of people experiencing financial scarcity could be affected through tunneling, cognitive load and time orientation.

#### Block 1—What is known about how financial scarcity affects self-control through tunneling, cognitive load and time orientation

The scarcity theory posits that attentional resources are allocated to financial demands, and that other information is often neglected, since the ability to focus and withhold information is restricted by available working memory capacity. This process is known as ‘tunneling’ [14]. When experiencing scarcity, reducing scarcity becomes a powerful objective, and suppresses other equally important, but less urgent objectives [14]. Since self-control requires focus and attention, and attentional capacity is consumed by financial demands, self-control execution becomes more difficult [58, 59]. There is growing evidence for attentional narrowing. People having too little financial resources shift their attention to thinking about money-related issues and to resolving these shortages [60–66]. However, the suggestion that the focus on urgent needs also leads to the neglect of other information, seems not to be fully supported by evidence [67, 68].

Financial scarcity is associated with constant worrying [14, 21, 63, 65, 67]. Continuous thoughts about insufficient resources and coping with demands, can be seen as cognitive load because it occupies cognitive capacity by holding information in working memory [69]. Cognitive load impairs decision-making of people experiencing scarcity, because it becomes harder to deploy executive functions such as selective attention^1^and self-control [21, 42, 58, 68, 73–75].

People are understood to behave more present oriented [21, 76] and less patient [64, 77–79] when experiencing financial scarcity versus a situation of affluence. Furthermore, people tend to discount future rewards more steeply when experiencing financial scarcity [67, 80–85]. These findings do not imply that people experiencing financial scarcity cannot consider future consequences. Several studies show that when the circumstances or future perspectives are more certain and predictable, people opt for later rewards [86–89].

#### Block 2—What is known about how tunneling, cognitive load and time orientation affect self-control in the context of dietary behavior

Managing attention, which is a more common construct in research on health behavior than ‘tunneling’, is required for self-control related imminent dietary decisions [26, 90–93]. Also, cognitive load has a critical role in self-regulatory eating behavior. A reduced cognitive capacity by applying cognitive load promotes unhealthy food choices [44, 94–98]. However, some studies report no effects of cognitive load [99, 100] and others report both disrupting and enhancing effects on dietary choices due to information processing biases [101–104]. For example, cognitive load may impede the capacity to recognize the immediate pleasure of giving into temptations [17, 70].

A present time orientation is related to more unhealthy dietary choices [105–110] and a future time orientation is related to preferences for healthier foods [111]. A present time orientation makes it more difficult to exert self-control in forthcoming challenging situations despite intentions to make healthy choices. This may be because the explicit immediate benefits of indulgence, and at the same time, the less tangible rewards of foregoing the instant gratification [112].

#### Premises on how dietary behavior of people experiencing financial scarcity could be affected through tunneling, cognitive load and time orientation

While hunger makes eating a recurring immediate demand, the planning needed for doing groceries, choosing a balanced menu taking account of nutritional values, and preparing healthy meals may not seem urgent when these compete with paying attention to solving financial dilemmas. Specifically in our contemporary obesogenic environment, scarcity induced tunneling may shift attention away from dietary quality demands, and cognitive load may impair attentional capacity. When attentional resources are burdened, it may be harder to pay attention to nutritional aspects of foods and stick to dietary goals, increasing the risk of indulgence. Furthermore, buying, preparing, and consuming healthy meals may be less rewarding for people experiencing financial scarcity because the costs in time, effort and expenditures are imminent, while the future effects of malnutritious diets are less certain.

## Methods

The review followed the PRISMA-ScR reporting guidelines for scoping reviews [113]. To identify relevant literature search strings were built together with an experienced information specialist of the University of Applied Sciences Utrecht to search PubMed, Embase, CINAHL, PsycINFO, Academic Search Complete and Web of Science. All search strings were published on searchRxiv [114–119]. Searching the six databases resulted in 10.856 papers of which 9.975 remained after removing duplicates in Zotero. The search was updated in July 2023 to include the most recent evidence.

In the identification phase we defined financial scarcity, tunneling, cognitive load, time orientation, and dietary behavior as key concepts and defined relevant synonyms.^2^ Synonyms for financial scarcity were, for example, financial dissatisfaction, financial stress, financial strain, socioeconomic status, and income. We defined food acquisition, food preparation, and food consumption as manifestations of dietary behavior. Synonyms for dietary behavior were, for example, nutritional behavior, food acquisition, food consumption, dietary choice, and dietary quality.

Papers which met the eligibility criteria for inclusion were studies in which the combination of financial scarcity and dietary behavior and one or more cognitive mechanisms were studied. Included were papers published in peer-reviewed journals presenting outcomes based on both quantitative and qualitative research methods, working papers and dissertations. No limitations on the year of publication or language were used.

Excluded were papers not reporting direct empirical evidence and papers reporting on food insecurity, because the accessibility of adequate food could alter cognitive processes and effects on dietary behavior. Also excluded were papers reporting on populations such as children and adolescents, because their executive functions are not fully developed, and their (perception of) financial circumstances and cognitive processes deviate from adults. Papers reporting on people suffering mental or physical illness, or with pathological dietary behavior, were excluded because cognitive processes may be affected by the illness.

The screening phase was conducted by the first author with ASReview, an open-source tool based on AI [120, 121]. With ASReview the references of all 9.975 papers were ranked and screened. ASReview is relatively new but has been used in several systematic reviews [122, 123] and was considered accurate and efficient [124, 125].

ASReview continuously ranks the references based on the assessment by the author of abstracts as potentially relevant or irrelevant, following the eligibility criteria. The SAFE-procedure^3^ was followed to screen the abstracts, which contains of four steps, with a different heuristic for the number of abstracts to screen in each step [126].^4^

**Fig. 2:**
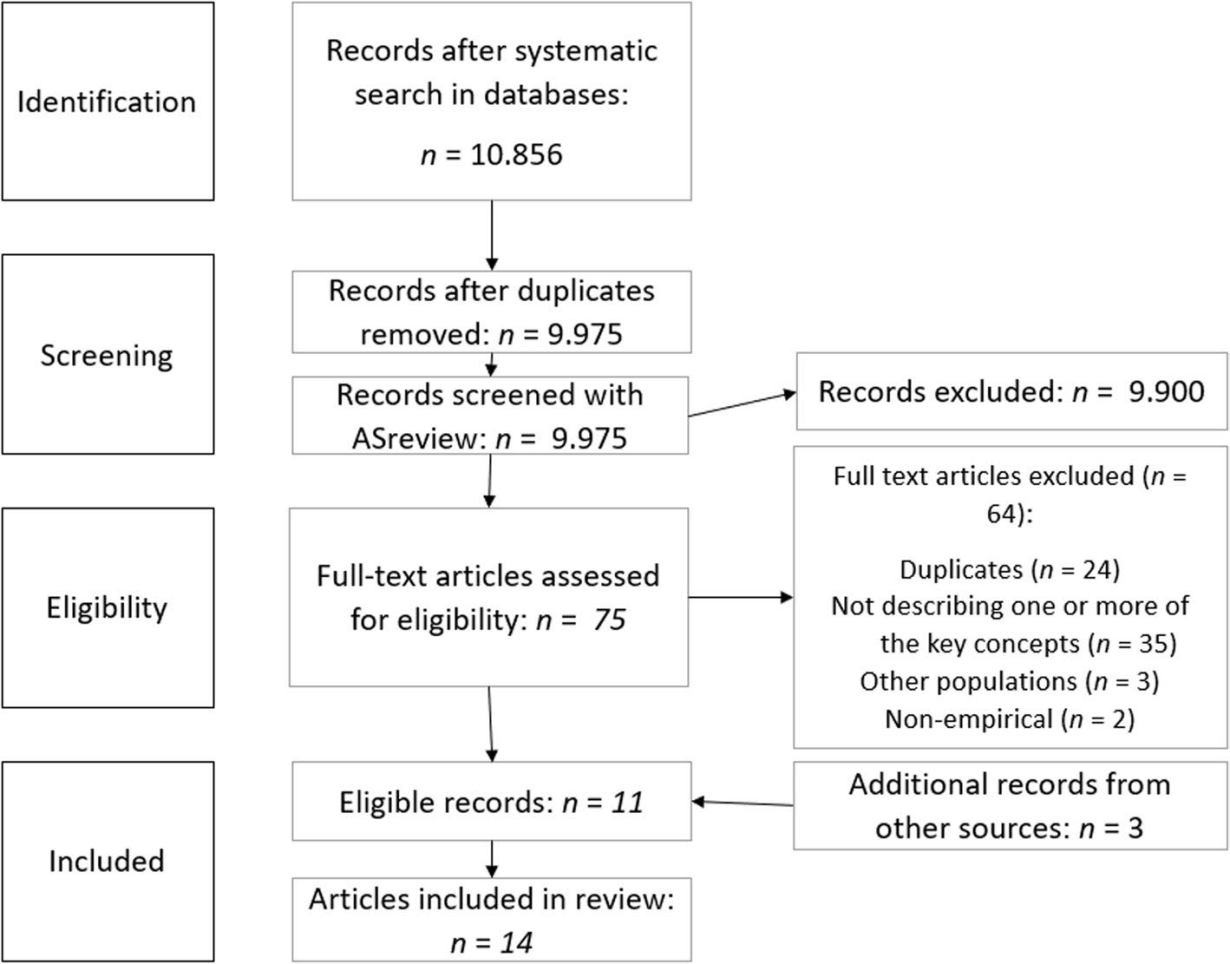
Selection process literature search

After concluding the SAFE-procedure 75 out of 9.975 papers were marked as potentially relevant. Then, the first author read the abstracts or the full texts of the 75 papers. A total of 11 papers met the eligibility criteria and 3 additional records were found. Thus, 14 full-text papers were included in the review (Fig. 2). Data abstraction was conducted by defining characteristics of the papers, such as a description of all variables, the research method, population, and conclusion.

## Results

Data-synthesis was conducted by grouping the included 14 articles by the mechanism they described: tunneling, cognitive load and/or time orientation and then by bringing together the main findings of the studies based on information about the study design, population, assessment of financial scarcity, main outcomes, and role of self-control. We also gave an indication of to what extent and how the main findings of the 14 articles provided support for scarcity theory (Appendix).

### How tunneling may affect dietary behavior when experiencing financial scarcity

Only one qualitative study brought forward insights of how tunneling and other financial scarcity mechanisms could impact dietary choices (see Table 1 in Appendix). Folta, Anyanwu, Pustz, Oslund, Penkert, & Wilson (2022) questioned 18 people about their food acquisition and found that participants invested considerable time and effort in obtaining foods at low costs and focused on costs above preferences and nutritional value. Thus, dietary choices did not seem to be driven by attentional neglect of nutritional facts or a lack of self-control. Rather, nutritional value was considered a lower priority in most cases, but exceptions were seen when participants experienced health problems [127]. The authors concluded this behavior was indicative of tunneling, triggering the higher prevalence of unhealthy dietary choices. The field experiment of Dominguez-Viera, Van den Berg, Handgraaf & Donovan (2023) supports these findings as most participants in their study were able to recall nutritional information when poverty concerns were manipulated, indicating attentional neglect was not an issue [128].

### How cognitive load may affect dietary behavior when experiencing financial scarcity

Evidence for the effects of cognitive load on dietary behavior of people experiencing financial scarcity stems from four experimental and two qualitative studies [95, 127–131] (see Table 2 in Appendix ). Self-control was only indirectly measured in two studies, and three studies pointed to other mechanisms than self-control as a driver for dietary choices. Cognitive load was shown to affect dietary choices when experiencing financial scarcity in two of the six studies.

Zimmerman & Shimoga (2014) suggested that people with lower socioeconomic positions could be more vulnerable to food advertising when cognitive load conditions are manipulated in comparison to people of higher socioeconomic positions, because cognitive load may magnify the effects of advertising malnutritious food. In their experiment participants of below-median-ses in high cognitive load conditions chose 84% more unhealthy snacks (with a mean of 143 more calories) when having watched food-advertising in comparison to those who didn’t watch food-advertising. The results among the above-median-ses were completely different; they chose 81% more of the healthy snacks after watching food-advertising. The researchers suggested that the daily hassles of living in poverty may resemble the experimentally induced effects of cognitive load [95]. Poulter, Eberhardt, Moore & Windgassen (2022) pointed out cognitive load as a risk factor for healthy dietary behavior. Cognitive load in the form of constant juggling and worrying in the context of limited budgets was reported by working adults living around the poverty threshold and was associated with reduced dietary quality. Grocery shopping coincided with substantive planning depending on costs and perishability. Besides the cognitive burden of job insecurity and low wages, the participants prioritized the dietary health needs of their children above their own, their health needs being the least important demand to be considered. These factors resulted in unhealthy diets, even when experiencing health problems and recognizing the importance of a balanced diet [130].

Four studies did not find an effect of cognitive load on dietary choices. Briers & Laporte (2013) manipulated financial satisfaction in a series of experiments with students to explore the interchangeability of the need for money with the need for energy dense foods. Participants who were financially dissatisfied ate significantly more high caloric food, especially when they regarded financial means as providing a feeling of security. Food energy was valued more in these conditions, and people experiencing financial dissatisfaction preferred higher-caloric foods more than financially satisfied people. The researchers imposed cognitive load with a digit recalling task in one of the experiments, but they found no moderation effect of cognitive load on the association between financial dissatisfaction and consumption of high caloric food [129]. Also, Folta, et al. (2022) didn’t find that cognitive load has a role in food choices in their qualitative study about the associations between financial scarcity and food acquisition. This may be related to the fact that the participants did not report high levels of stress and had abundant time to consider their purchases [127]. In their field-experiment in Mexico-city Dominguez-Viera et al. (2023) induced poverty related concerns on primary household shoppers and found that this reduced the willingness to pay for a healthy variant of packaged bread. Increased stress but not cognitive load mediated this association. Furthermore, the manipulation did not alter the ability to recall nutritional and health aspects [128]. In an online experiment of Pechey & Marteau (2018) in which main and interaction effects of socioeconomic status and cognitive load with the availability of healthier versus less healthy snacks on food choice were tested, no effects were perceived [131].

### How time orientation may affect dietary behavior when experiencing financial scarcity

Eight studies have investigated the associations between dietary acquisition and consumption and time orientation of participants in financial scarcity conditions (Appendix, Table 3). Time orientation is often measured by the extent of discounting future rewards, which means the preference for smaller immediate rewards versus delayed but larger rewards, for example, choosing 75 dollars now versus 125 dollars in a year. Four studies used an experimental design, two a cross-sectional design and two a qualitative design. Self-control was only mentioned in the qualitative studies. Four studies are indicative of an effect of time orientation on dietary behavior in situations of financial scarcity. The other four studies did not provide evidence for an effect: however, two of these assessed income in their design, which is a rather distal and objective proxy for financial scarcity.

Mellis, Athamneh, Stein, Sze, Epstein & Bickel (2018) manipulated a situation of acute scarcity by letting participants with obesity read a narrative about being let down and having to spend all savings to move to another part of the country. In comparison to the income-neutral event, in the scarcity condition the participants showed a declined ability to delay gratification and a higher demand for fast food [132]. Laran & Salerno (2013) demonstrated that when participants were primed to think of environmental harshness coupled with thoughts on short duration, relatively more participants would choose high filling foods as opposed to participants thinking of harshness coupled with thought on longer duration. This suggests that when primed to think about the present in environmentally harsh conditions people will be more prone to choose unhealthy foods. Interestingly, when people were primed to think of the future in the same environmentally harsh condition, a higher proportion chose the healthier variant of the proposed foods. The authors suggest that a present time orientation is related to resource seeking (in money or food) not in pleasure seeking. Choosing high caloric food may therefore be considered functional instead of the consequence of a lack of self-control [133]. The effects of present time orientation were also self-reported in a qualitative study of Kaplan, Madden, Mijanovich & Purcaro (2013). When asked about the processes contributing to overeating, 56 adults living in a deprived neighborhood reported high levels of chronic stress, partly due to finances. The participants indicated that these stressors resulted in disinhibited eating and that discounting the future and self-control problems were two of the processes driving the behavior. First, the lack of future perspectives made it seem pointless to invest time and energy in eating healthily and second, the participants experienced so many daily difficulties that they felt depleted and therefore were less resistant to temptations, even while recognizing the risks [134]. These daily stressors could also be an indication of tunneling or cognitive load. Another qualitative study supported these findings. Dumas, Robitaille & Jette (2014) interviewed 15 Canadian women living in underprivileged areas, most of them unemployed. The women reported struggling with financial responsibilities and prioritizing imminent needs instead of future health. The authors indicated that a present bias explained choices regarding food acquisition and weight management that are inconsistent with dietary recommendations [135].

Other studies have contradictory outcomes. A study of Sze, Stein, Bickel, Paluch & Epstein (2017) showed that when primed to think about personalized positive future events, participants valued future health benefits more, even when experiencing financial scarcity. Also, in the scarcity condition participants showed higher delay discounting. However, participants showed lower demand intensity in the scarcity condition [136]. In an experiment of Stein, Craft, Paluch, Gatchalian, Greenawald, Quattrin, Mastrandea, Epstein & Bickel (2021) the effect of scarcity on time orientation was seen, but they did not find a subsequent effect on dietary choices [137]. Appelhans, Tangney, French, Crane & Wang (2019) executed a household food shopper study in which the food purchases of primary household shoppers were registered for 14 days, and they measured discounting with a monetary reward task. They found discounting rates to be positively associated with buying more malnutritious foods. This association, however, was not moderated by a poverty-to-income ratio [107]. In a survey study, Shuval, Stoklosa, Pachucki, Yaroch, Drope & Harding (2016) demonstrated that respondents with a present time perspective consumed fast food more frequently. A significant relationship between time preference and fast food consumption was found only in the middle-income category [138].

### Synthesis of results

**Fig. 3:**
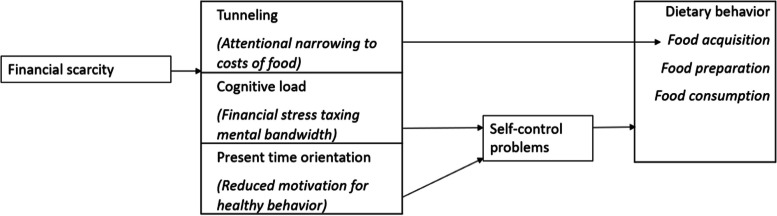
Synthesis of results

The evidence presented points to various potentially disrupting effects of financial scarcity on dietary behavior via tunneling, cognitive load and time orientation. Tunneling seems to be restricted to attentional narrowing through focusing on the costs of food in food acquisition. Evidence was not found for attentional neglect nor for effects of tunneling on food preparation and food consumption. A focus on the costs of food is indicative of reasoned rather than impulsive decision-making in food acquisition, suggesting that self-control in eating behavior was not affected by tunneling. When cognitive load is explained as the burden of chronic stress that accompanies financial problems or insecurities, it could impair dietary behavior because it captures attentional capacity. Evidence of the latter is, however, inconclusive, since focusing on financial problems could also distract people from palatable food cues. Finally, financial scarcity could increase discounting of health benefits. Evidence suggests that when burdened with financial strain, the future consequences of dietary choices are perhaps discarded because of a perceived lack of prospect in the long term, suggesting a shift in priorities to immediate gratification and a loss of motivation for following up recommendations for healthy diets. Figure 3 visualizes our synthesis.

## Discussion

### Summary of evidence

Our scoping review leads to several findings. First, the single study included on scarcity induced tunneling suggested that costs of food products are prioritized in food acquisition, suggesting a shift in attention, not a loss of self-control per se. Therefore, we cannot assume that when experiencing financial scarcity, people ignore or are unaware of information outside their ‘tunnels’, such as an advertisement for, or the nutritional value of (un)healthy products. Second, cognitive load may interfere with selective attention and adhering to dietary goals. When experiencing scarcity, cognitive load occupies mental resources needed for executive functioning, possibly interfering with self-control. However, research on the association between cognitive load and food choices is not unequivocal. Third, financial scarcity affects time orientation. Attention shifts not only to the problem at hand, but also to the present, resulting in a present time orientation. This may also affect the way people perceive the future consequences of dietary choices. Engaging in healthy dietary behavior may seem less urgent, which reduces the motivation to stick with long-term goals related to healthy eating behavior.

### Directions for future research

We reported some qualitative studies involving people living in poverty which showed how the mechanisms of tunneling, cognitive load and time orientation coincided and affected healthy diets. However, we could not make a firm conclusion about how these cognitive mechanisms may interact with each other. Since tunneling shifts attention and cognitive load diminishes attentional capacity, such interaction effects can be a promising new line of inquiries.

Furthermore, many participants in the qualitative studies reported that having to deal with financial problems day after day made it difficult to focus on long-term health goals. Therefore, research into the role of time orientation may provide further insights in understanding how perceptions of the present and future affect dietary behavior when experiencing financial scarcity. A possible angle may be looking at the extent to which this process is value-driven or awareness-driven [139]. When a person considers the future as less reliable, future outcomes will lose their value. This may lead to a decline in motivation to eat healthily. But a person may also simply not be aware of the future, because it is not in their tunnel. In this sense, preferring immediate rewards may appear as impulsive behavior, but in fact is rational behavior that is explained by the fact that people have actual constraints, such as liquidity problems [140, 141].

Further research is needed to explore how dietary behavior interventions can restore and increase motivation for following up recommendations for healthy diets by people experiencing financial scarcity. Even when people experiencing financial scarcity know that healthy eating behavior is important to prevent chronic illnesses, and they express a will to pursue high diet quality [142], doing so requires constant attention and self-control. Interventions that reduce financial scarcity and help build habits in healthy dietary patterns may relieve and circumvent the strain on mental bandwidth in these situations [143, 144].

Evidence for the nature of associations and the extent to which cognitive mechanisms affect dietary behavior of people experiencing financial scarcity is still very limited. A first step we recommend is building theoretical frameworks that explain when, how and why cognitive mechanisms elicited in a financial scarcity mindset affect self-control related dietary choices. These frameworks should enhance the use of carefully defined constructs and describe the underlying behavioral and psychological mechanisms more precisely. Secondly, we recommend experiments and field studies to elaborate on the strength of the associations between financial scarcity and dietary behavior, through cognitive mechanisms. Field studies should be conducted to better represent the enduring and evasive nature of financial scarcity. Finally, we recommend qualitative studies with people experiencing financial scarcity that help understand the drivers and obstacles in goal-directed behavior for maintaining a healthy diet.

### Strength and limitations

Our synthesis of the literature fits to current theoretical approaches as life history theory [145–148] and reinforcer pathology [149–151] that suggest that in situations of financial scarcity dietary behavior could be affected through shifts in attention and priorities, making dietary choices more adaptive to or logical consequences of energy-consuming insecure and unstable situations [152, 153], suggesting that dietary decisions of people experiencing financial scarcity could be both instrumental and stress-driven.

The limited number of studies prevent us from making firm conclusions on the role of tunneling, cognitive load and time orientation, elicited by financial scarcity, on dietary behavior. Results should be interpreted with caution, because there are limitations in the generalization of the research outcomes of the included papers, due to the large variability of studies in terms of operationalization of the key concepts and the design of the studies.

First, recent studies did not use a single concept of financial scarcity. The context of financial scarcity differs, as it can be evoked by relative deprivation [154], income uncertainty [60], negative income shocks [132, 136], or anticipated future shortcoming of available financial resources [84] and poverty-related cues [155, 156]. Furthermore, to explain the behavioral consequences of financial scarcity, using income as a measure may not suffice. The lack of a single definition or operationalization applies to the concepts of self-control, tunneling, cognitive load and time orientation as well, possibly interfering with the validity of research outcomes [157, 158].

Second, our review included studies using experimental designs which have their limitations. Financial scarcity can be much more evasive and enduring than when elicited temporarily. Cognitive load is also often temporarily induced on study participants. Furthermore, in experiments considering the role of attention on eating behavior, people (whether ‘rich’ or ‘poor’) usually get a choice in what and how much they want to eat. In the real world, people with financial strains are limited by their budgets and they will have to choose (long) before consumption. Therefore, laboratory studies are likely to underestimate the consequences of financial scarcity on eating patterns.

## Conclusions

The scarcity theory has brought a new perspective on the impact of financial scarcity on decision-making and behavior. We provide a thorough basis for the current assumption that a focus on the here and now interferes with diet quality when experiencing financial scarcity. Empirical research testing this assumption is relatively new and up till now very limited. In synthesizing the evidence, the scoping review addresses the potential relevance of cognitive processes driving dietary behavior-related decisions in financially demanding situations.

## Appendix

**Table 1:** Literature overview of the impact of financial scarcity induced tunneling on dietary behavior

**References**	**Study design & population & assessment of financial scarcity**	**Main outcome**	**Role of self-control & indication of support for scarcity theory**
Folta, Anyanwu, Pustz, Oslund, Penkert & Wilson (2022) [127]	Accompanied shop, interviews, participant driven photo elicitation Men and women (*n* = 18) Participants meeting federal guidelines for poverty	The costs of food and preferences are prioritized above nutritional value when acquiring groceries	Self-control was not measured. Dietary choices do not seem to be impulsive in relative time abundant conditions Behavior indicative of tunneling

**Table 2:** Literature overview of the impact of financial scarcity induced cognitive load on dietary behavior

References	Study design & population & assessment of financial scarcity	Main outcome	Role of self-control & indication of support for scarcity theory
Folta, Anyanwu, Pustz, Oslund, Penkert & Wilson (2022) [127]	Accompanied shop, interviews, participant driven photo elicitation. Men and women (*n* = 18) Participants meeting federal guidelines for poverty	The costs of food and preferences are prioritized above nutritional value when acquiring groceries. Participants did not report indicators of cognitive load	Self-control was not measured. Dietary choices do not seem to be impulsive in relative time abundant conditions No evidence for (effects of) cognitive load
Zimmerman & Shimoga (2014) [95]	Experiment: 2 × 2 factorial design. Effects of advertising and cognitive load on number of snacks chosen and total in calories Students (*n* = 351) Stratified by parental ses by proxy of parental zip code	Low ses-individuals are more susceptible to the effects of advertising in conditions of high-cognitive load than high-ses individuals, leading to a large increase in the number of snacks chosen and calories consumed	Self-control was not measured Authors suggest cognitive load experienced when living in poverty may explain sensitivity to food marketing for low-ses individuals
Briers & Laporte (2013) [129]	Five lab experiments. Effects of financial (dis)satisfaction on food preferences and consumption. Students (*n* = 63) Manipulation of financial satisfaction	Financial dissatisfaction increases motivation to eat high caloric foods. No main or interaction effect of cognitive load on calories eaten	Food overconsumption may reflect a different mechanism than self-control. Financial dissatisfaction may lead to automatic, non-conscious preferences for high caloric foods No evidence for effects of cognitive load
Poulter, Eberhardt, Moore & Windgassen (2022) [130]	Semi-structured interviews. Women (*n* = 5) and men (*n* = 1) In-work poverty: financial resources close to poverty-threshold	Participants described cognitive load as a constant and uncontrollable process, requiring cognitive capacity and impacting mental health, relations, and sleep Health needs were considered the least priority due to financial scarcity, mental exhaustion, and guilt	Food acquisition does not seem to be impulsive, as participants described it as a task requiring a lot of planning. Dietary choices were affected by economic and time factors, rather than health, preferences, or lack of self-control Cognitive load is seen as risk factor, affecting the perceived capability, opportunity, and motivation to perform health behaviors
Pechey & Marteau (2018) [131]	Online experiment. Effects of the number of healthier and less healthy snack foods on food choices, including moderation effects of cognitive load and ses Men and women (*n* = 1.509) Ses measured by occupation, education, household income and index of multiple deprivation	No main or interaction effects of cognitive load on food choice. No effects of ses on food choice were found. Food appeal but not response inhibition mediated differences in food choice by ses-groups	Self-control was not measured No evidence for effects of cognitive load
Dominguez-Viera, Van den Berg, Handgraaf & Donovan (2023) [128]	Field experiment, 2 × 2 factorial design. Effects of nutrition information and poverty concern on willingness to pay for healthier packaged bread, richer in protein and fiber and less sodium Men and women (*n* = 423) Three low-income municipalities of Mexico City and induced poverty concerns	Poverty related concern increases stress not cognitive load. Willingness to pay for the healthier variant of the bread was affected by poverty concerns, via increased stress. Willingness to pay did not differ between income groups. Cognitive load was not a mediator Attention to provided information on nutritional value did not differ by poverty concern and/or income	Self-control was not measured No evidence for effects of cognitive load Manipulating poverty related concerns did not seem to increase attentional neglect

**Table 3:** Literature overview of the impact of financial scarcity induced time orientation on dietary behavior

References	Study design & population & assessment of financial scarcity	Main outcome	Role of self-control & indication of support for scarcity theory
Mellis, Athamneh, Stein, Sze, Epstein & Bickel (2018) [132]	Online experiment. Effects of negative income shock on discounting money and food and on purchasing fast food and water Men and women with obesity (*n* = 120) Negative income shock was manipulated through a narrative	Negative income shock elicited greater discounting of money and food and in this condition participants showed a higher intensity of demand (consumption unconstrained by price) for fast food not water	Self-control was not measured Discounting increased in situations of financial scarcity and elicited unhealthy choices
Laran & Salerno (2013) [133]	Experiment 2 = 3 × 2 between subjects design. Effect of harshness and resources provided (1 dollar) on food choice Students (*n* = 238) Environmental harshness was primed by showing participants words associated with harshness Experiment 3 = 2 × 2 between subjects design. Effects of harshness condition and duration on food choice. Students (*n* = 144)	Experiment 2. When given resources participants were less likely to choose the food that was perceived to be more filling Experiment 3. When primed to think of a harsh condition coupled with a short duration, more participants chose food that was perceived to be more filling, than when primed to think of the same condition coupled with a long duration	Self-control was not measured Experiment 2. When resources were provided participants did not choose high calorie foods in the harsh condition Experiment 3. Results suggest an effect of time horizon on food choice. When focused on the present, participants made more unhealthy choices in harsh conditions
Stein, Craft, Paluch, Gatchalian, Greenawald, Quattrin, Mastrandrea, Epstein & Bickel (2021) [137]	Experiment. Effects of episodic future thinking and economic scarcity on discounting and demand for fast food by a food purchase task Men and women at risk for diabetes (*n* = 78) Economic scarcity was manipulated through a narrative	Scarcity increased discounting. No effect of scarcity on food demand	Self-control was not measured Discounting increased in situations of financial scarcity but no effects of discounting on food choices in these situations were found
Sze, Stein, Bickel, Paluch & Epstein (2017) [136]	Online experiment, 2 × 3 factorial design. Effects of episodic future thinking and negative income shock on discounting and demand for fast food by a food purchase task Men and women (*n* = 204) Negative income shock was manipulated through a narrative	Negative income shock elicited greater discounting of money. Participants showed lower demand intensity after reading the scarcity narrative. Episodic future thinking decreased discounting and demand for fast food in negative income shock condition and in absence of the scarcity condition	Self-control was not measured Discounting increased in situations of financial scarcity but no (expected) effects of discounting on food choices in these situations were found
Kaplan, Madden, Mijanovich & Purcaro (2013) [134]	7 focus groups on the perception of stress and its relationship to health and health behavior Men and women (*n* = 56) Residents from a low-income community in New York	Participants explained the relationship between (financial) stress and unhealthy (eating) behavior (overeating, erratic eating, eating too much high fat foods or forgetting to eat) through self-medication, adaptive behavior, discounting the future, loss of willpower and competing priorities. Participants mentioned that they were not motivated to engage in healthy behavior. Investing in healthy behavior seemed pointless considering their future perspectives Participants mentioned other priorities and a lack of time to invest in or pay attention to healthy behavior	Participants mentioned that (financial) stress depletes will-power even when aware that unhealthy (eating) behavior impairs health Discounting was an explanation for unhealthy dietary choices Behavior also indicative of tunneling or cognitive load
Appelhans, Tangney, French, Crane & Wang (2019) [107]	SHoPPER study: cross-sectional study. Choice task in combination with analysis of food receipts. Relation between discounting and healthfulness of food purchases Men and women (*n* = 202) Poverty-to-income ratio	Steeper discounting was related to lower overall healthy eating index scores (HEI-2015) and a higher energy density. Poverty-to-income ratio did not moderate the association between discounting and food purchases	Self-control was not measured No effects of financial scarcity condition on the association between discounting on food choices were found
Shuval, Stoklosa, Pachucki, Yaroch, Drope & Harding (2016) [138]	Survey study. Future time perspective and frequency of fast food and full-service restaurant consumption Men and women (*n* = 5.871) Annual income	High future time perspective is related to less frequent fast food intake (not full-service restaurant intake). There was not an interaction effect of income and time preference on frequency of fast food consumption A significant relationship between time preference and fast food intake was only found in the middle-income group	Self-control was not measured No interactions effects of time orientation and income on food choices were found
Dumas, Robitaille & Jette (2014) [135]	In-depth interviews. Sociocultural factors underlying dispositions towards health practices Young and underprivileged women (*n* = 15)	Financial responsibilities and focus on present needs were drivers of current food acquisition and weight management. The participants did not think of the future, but instead prioritized economic stability, family needs, or current illnesses	A lack of self-control was mentioned by some of the women A present bias was an explanation for unhealthy dietary choices Behavior is also indicative of tunneling, but could be instrumental since investing in health was seen as strategy when planning for a better future

## Data Availability

Not applicable. The complete search strategy is available from the corresponding author on reasonable request.

## Abbreviations

AI: Artificial Intelligence
SAFE-procedure: Screening Apply Find and Evaluate
SES: Socioeconomic Status

## Footnotes

1 In this sense, we see ‘selective attention’ as an executive function and distinguish it from tunneling or narrowing attention. We see tunneling as an instrumental process, regardless of the presence of cognitive load. We recognize that apart from tunneling, cognitive load may affect the ability to pay selective attention to a certain task or goal [70–72].

2 We were interested in papers on the three key concepts of tunneling, cognitive load and time orientation. Therefore, we did not include self-control as a separate obligatory concept in the search string, to prevent a too narrow search string and assuming that relevant papers on the three key concepts would include papers on self-control.

3 SAFE is an acronym which stands for the four steps of Screening, Apply (with active learning based on TF IDF Naïve Bayes model), Find (with deep learning based on Sentence BERT model) and Evaluate. The Evaluation phase can be seen as a reliability check.

4 The SAFE-procedure was developed by Boetje en Van de Schoot to improve the efficiency of the screening process. The procedure minimizes the risk of missing potentially relevant papers and reduces the number of irrelevant abstracts that need to be screened. This means that after screening the advised number of abstracts, chances are very low that when proceeding the screening, potentially relevant papers are found. A publication on the procedure is available as preprint [126].
